# Severe Hemoperitoneum due to Ovarian Bleeding after Transvaginal Oocyte Retrieval with Surgical Management: A Retrospective Analysis and Comprehensive Review of the Literature

**DOI:** 10.3390/medicina59020307

**Published:** 2023-02-07

**Authors:** Jelena Stojnic, Jovan Bila, Lidija Tulic, Jelena Micic, Mladen Andjic, Miljan Pupovac, Ivana Likic Ladjevic, Tatijana Tosic, Jelena Dotlic

**Affiliations:** 1 Faculty of Medicine, University of Belgrade, Dr Subotica Starijeg 8, 11000 Belgrade, Serbia; 2 Clinic of Obstetrics and Gynecology, University Clinical Centre of Serbia, Dr Koste Todorovica 26, 11000 Belgrade, Serbia

**Keywords:** hemoperitoneum, IVF/ICSI, oocyte retrieval, bleeding, ovary, hemorrhage, complications, hemostasis

## Abstract

*Background and Objectives*: Severe hemoperitoneum of ovarian bleeding origin is a rare but potentially life-threatening complication of transvaginal oocyte retrieval (TVOR) procedure. The study aimed to present a case series of surgically managed patients from our clinic with hemoperitoneum caused by ovarian bleeding after TVOR, as well as to perform a comprehensive literature review in order to summarize and analyze all published cases with this condition and their management. *Materials and Methods*: The data of 2939 patients, who underwent TVOR procedures for IVF/ICSI (in vitro fertilization, intracytoplasmic sperm injection) in our clinic between 2010 and 2021 were reviewed. Moreover, a systemic literature search was performed. Main outcome measures from the pooled analysis were incidence and risk factors, type of surgery, intraoperative finding and intervention leading to hemostasis. *Results*: In our Clinic 4 (0.136%), cases of hemoperitoneum due to ovarian bleeding were surgically managed. Moreover, 39 cases from 18 studies reported in the literature were identified. No risk factors besides lean women with PCOS were identified. In the pooled analysis, the bleeding symptoms appeared in 58.1% of patients within eight hours after TVOR and cumulatively in 81.4% cases during the 24 h after TVOR. The average time from TVOR to surgery was 27.19 ± 53.25 h. Hemostasis was mostly established using electrocoagulation, although few cases of ovariectomy were also reported. Embryo transfer at 60% of cases was postponed and embryos cryopreserved. *Conclusions*: Severe hemoperitoneum due to ovarian bleeding after TVOR is a rare event that should be treated by techniques of minimally invasive surgery whenever possible. Protocols should be developed to enable optimal management strategies for infertility patients. Embryos obtained should be cryopreserved.

## 1. Introduction

Since “sonographically controlled vaginal culdocentesis” was first described by Gleicher et al. in 1983, the technique of ultrasound-guided transvaginal oocyte retrieval (TVOR) has become the method of choice in in vitro fertilization (IVF) and intracytoplasmic sperm injection (ICSI) for oocyte collection, and about 99% of TVOR interventions are performed this way [1,2,3]. However, introducing a sharp needle through the vaginal wall and into the ovary under ultrasound guidance and monitoring can cause damage to the vagina, parametric tissues [4,5,6], and ovary-adjacent pelvic organs, such as the uterus, bladder, urethra [7,8,9,10], bowels [11,12,13], and large pelvic blood vessels [14,15,16,17].

Published data about hemoperitoneums of ovarian origin after TVOR are scarce and are mainly presented in the form of case reports or limited retrospective studies [18,19,20,21,22,23,24,25]. Few larger studies have reported the frequency of hemoperitoneums after TVOR but without specifications for each patient and no cause of bleeding clearly described [5,6,26]. Underreporting of this potentially very serious complication may be due to the rarity of the event and the fact that reproductive specialists’ main focus is the efficiency of the procedure and perinatal outcomes rather than potential complications.

A certain amount of blood can routinely be seen after TVOR in the peritoneal cavity. This is considered normal during intervention and in the post-intervention period and has no clinical significance if there are no signs of active bleeding or hemodynamic instability. Dessole et al. estimated that for a non-complicated TVOR, normal blood loss is about 230 mL in the first 24 h after intervention [27]. Yih et al. [28] detected non-significant changes in hematocrit levels in several subsequent complete blood counts before and after TVOR [28]. In one study, the quantity of blood loss in 150 women after TVOR was subjected to analysis through serial measurement immediately after TVOR, at 4 to 6 h and 72 h later, by analyzing the complete blood count and ultrasonographic appearance of free liquid (blood). No significant changes were found [29]. 

Previously published pooled analyses have shown that the incidence of severe hemoperitoneums caused by ovarian bleeding after TVOR ranges from 0.08% [18,25] to 0.22% [21]. Some authors have also suggested that with modern technologies, the incidence of oocyte retrievals with severe hemoperitoneums of all bleeding origins is decreasing [26]. 

The symptoms of hemoperitoneum of ovarian origin tend to become apparent several hours after the patient is discharged from the IVF unit; thus, the diagnosis and therapy are in the hands of emergency gynecologists. Protocols of steps and procedures in such cases have not yet been established; thus, emergency gynecologists at the tertiary care level are treating this problem as they would any other hemoperitoneum. If severe hemoperitoneum after TVOR develops, a blood transfusion and emergency surgery to obtain hemostasis are required. If the observation period is long with delayed surgery, complete oophorectomy or partial ovary resection may be needed for hemostasis [25]. Oophorectomy as a solution for hemostasis is not a desirable option for infertility patients, nor is a prolonged observation period with possible negative reproductive and life-threatening consequences.

The study aim was to present a series of patients who had a hemoperitoneum caused by ovarian bleeding after TVOR from our clinic, from 2010 to 2021, as well as to perform a comprehensive literature review in order to summarize and analyze all published cases with this condition, to enable optimal management strategies for such complications.

## 2. Materials and Methods

All 2939 patients submitted to IVF/ICSI procedures at the clinic at Ob/Gyn University Clinical Center of Serbia during the past 12 years (1 January 2010 to 31 December 2021) were retrospectively analyzed. Out of them, those who had an emergency surgery in our clinic due to a severe hemoperitoneum caused by ovarian bleeding after TVOR were included in the study. 

The study was approved by the Ethics committee of the University Clinical Centre of Serbia (number 922/2). The study conformed to the Helsinki Declaration, the Committee on Publication Ethics (COPE) guidelines (http://publicationethics.org; accessed on 1 August 2022), and the Reporting of Studies Conducted Using Observational Routinely collected Health Data (RECORD) statement, available through the Enhancing the Quality and Transparency of Health Research (EQUATOR) network (www.equator-network.org; accessed on 1 August 2022). All patients that were involved in the study were retrospectively contacted, and they gave their consent both for the study as well as for publication of their data. The collected data were anonymized, taking into the account the observational character of the study, without personal data, that could allow for the identification of the patient.

Hemoperitoneum was established according to standard signs and symptoms such as diffuse pelvic and abdominal pain, shoulder pain, dizziness, bloating, nausea, vomiting, weakness, cold sweeting, dyspnea, tachycardia and hypotension [25]. Severe hemoperitoneum was preoperatively confirmed by a significant decrease in hematocrit and hemoglobin levels with hemodynamic instability, and a medium to large volume (depth ≥ 5 cm) of unclear fluid in the pelvis was demonstrated by ultrasound examination and proven ovarian bleeding during surgery or with the use of computer tomography (CT). 

For patients included in the study, we noted general data such as age and body mass index, infertility cause, previous surgery (ovarian) and comorbidities that could have increased risks for bleeding. In addition, the number of previous IVF attempts and parity with possible deliveries were recorded. Laboratory findings before TVOR (on the triggering day) and before and after surgical treatment were also registered (hemoglobin and hematocrit levels). Duration of hospital stay after surgery was noted also. 

The patients underwent controlled ovarian stimulation using standard luteal phase long protocol with GnRh (gonadotrophin releasing hormone) agonist, dominantly about 80% of all COS to 2012 or flexible GnRh antagonist protocol, with about 87% of all protocols, dominant from 2014 in our IVF Centre. With the use of GnRh antagonists protocol, triggering agent of final oocyte maturation is performed with human chorionic gonadotrophin (HCG) usually or GnRh agonist under special circumstances (the total number of follicles, the mean serum estradiol concentration). We evaluated the following parameters regarding ovulation stimulation such as type of stimulation protocols, duration in days of ovarian stimulation, the total dose of gonadotropins used, and maximum estrogen serum levels on the triggering day.

TVOR was performed 35 h after the human chorionic gonadotropin injection had been given. The intervention was performed under general anesthesia, intravenously with midazolam, fentanyl and propofol, and with assisted mask ventilation with oxygen and inhalatory anesthetic if needed. Vaginal preparation included cleansing with isotonic saline solution. Prophylactic antibiotic was routinely given, Gentamycin a 120 mg in one dose immediately after intervention to all the patients. The follicles were retrieved by sequential puncture without reinsertion of the needle, 17-gauge diameter, single-lumen, through vaginal wall, and one puncture site was made on each side, using negative pressure of 140 mmHg. All follicles along the same axis were punctured one by one, without reinserting the needle. If the oocyte was not recovered in the first attempt of aspiration, the follicle was flushed with medium up to three times. At the end of the procedure, the intraperitoneal space and Douglas pouch were checked in and investigated on ultrasound for traces of bleeding, hematoma formation or fluid accumulation. After the procedure, the vagina was examined with a speculum for traces of bleeding, and compressional hemostasis was applied if it was necessary or suturing to manage bleeding. The patients were observed at least 4 to 6 h after the intervention, closely monitoring for signs of complications, checking the blood pressure, pulse and vital signs and presence of abdominal pain.

After the TVOR procedure, all patients were administered intramuscular progesterone at 250 mcg every second day and had intravaginal micronized progesterone (200 mg soft capsule) three times per day for luteal phase support. The patients were administered beta HCG test 14 days after embryo transfer. Positive tests were recorded. Clinical pregnancy was determined as presence of gestational sac with embryo presenting the heart motion up to 8th week of gestation.

Parameters of TVOR such as the number of retrieved oocytes, the needle used for TVOR and the suction pressure used, sedation or general anesthesia during TVOR, the time interval between TVOR and the onset of bleeding and hemoperitoneum symptoms, the time interval between TVOR and surgical intervention were assessed. 

Regarding surgery, we evaluated the approach (laparoscopy/laparotomy), type of intervention performed for hemostasis, the ovary as a source of bleeding (type of lesion), blood volume in abdominal cavity, administered blood transfusions and overall outcome (good/adverse). Finally, we analyzed the outcome of ART (abolished/delayed/proceeded). Embryos can be transferred in the fresh cycle after the surgery, or cryopreserved and transferred in another cycles, which is more often. 

A comprehensive literature review and the pooled analysis of all presented patients with hemoperitoneum caused by ovarian bleeding after TVOR was also performed. This methodology was applied in some other studies so that summary and comparison of rare published data could be achieved [25,30], the definition of severe hemoperitoneum caused by ovarian bleeding after TVOR was defined earlier, and criteria regarding ovarian source of bleeding had to be strictly proven, either by operation or CT. We searched the following electronic databases for studies PubMed, Scopus, Web of science, Medline and EMBASE (search date 8 August 2022) using different combinations of the following key words: ART (assisted reproductive technology), IVF/ICSI, oocyte retrieval, complication, bleeding, hemorrhage, hemoperitoneum, surgery. Studies were included if they were freely accessible complete reports in English. Only data published and available in the manuscripts were used for further analysis, and no additional information was sought from authors. The full text papers were evaluated individually by two authors, and the third author revised them. Studies were included in the analysis if they presented characteristics of each patient, TVOR and surgery in full details. Studies that met criteria were excluded if the source of bleeding was unknown or not mentioned, or from the other location except ovary such as urinary system origin, and the result of the big vessels lesion. Otherwise, studies regarding TVAO complications were taken into consideration only for general comparison of findings. The final eligibility decision was made with consent from two authors, who evaluated the publications, and possible disagreements were solved through a constructive meeting. The definitions of conditions and inclusion/exclusion criteria had to be the same for our patients as well as for those presented in the assessed literature. We noted the same parameters regarding characteristics of patients, ART and TVOR procedures and surgery for patients previously presented in the literature as for our patients. Finally, data extracted from the literature were analyzed together with data of our patients.

All pre- and postoperative data were statistically analyzed using methods of descriptive (mean, standard deviation, median, range, frequencies, percent) and analytical statistics (Hi square and *T* test). Analyses were performed first just for our patients and then for the pooled sample. The values *p* < 0.05 are accepted as significant. Analyses were performed using SPSS for Windows version 22 (SPSS, Inc., Chicago, IL, USA).

## 3. Results

During the examined past twelve years, 2939 oocyte retrieval procedures were performed in our Clinic. There were four cases (0.14%) of hemoperitoneum caused by ovarian bleeding after TVOR. Patients’ characteristics are presented in Table 1.

### 3.1. Case One

A 38-year-old patient was treated in our unit for secondary infertility due to tubal obstruction, with a history of laparotomy with right salpingectomy for ectopic pregnancy 16 years prior and for right ovarian cystectomy and plastic repair of the right fallopian tube 15 years prior. The patient had undergone one previous IVF attempt but had not achieved pregnancy. In her medical history, there were no other problems, and she had an anti-Mullerian hormone (AMH) level of 1.47 ng/mL. Controlled ovarian stimulation (COS) was conducted using a long GnRh agonist protocol, with 25 ampoules of GnRh agonist and 2400 IU recombinant follicle stimulating hormone (rFSH) for 11 days. The peak estradiol level on the triggering day was 3176 pmol/L, with retrieval of six oocytes. The patient returned to the emergency room 18 h after TVOR, presenting with hemodynamic instability, diffuse abdominal pain, dizziness, and severe weakness. The ultrasound revealed a large amount of intraperitoneal fluid with a pocket at the Douglas pouch, with a diameter of about 8–9 cm. The emergency laparotomy was performed, revealing a hemoperitoneum of about 1200 mL of blood and profuse bleeding from the right ovary laceration, about 2 cm in length. Hemostasis was achieved through multiple sutures and repair of the right ovary. In total, the patient received four units of blood and two units of fresh frozen plasma. The freeze embryo transfer was performed 1.5 years later with three cleavage embryos on day 3, but no pregnancy was achieved.

### 3.2. Case Two

A 34-year-old patient, gravida 0, para 0, underwent her first IVF attempt for unexplained infertility. She had had two previous intrauterine insemination (IUI) procedures performed. Her AMH level was 1.75 ng/mL, and no medical problems were reported. The GnRh antagonist protocol was employed with a total dose of rFSH 2025 IU over 9 days of stimulation, and peak estradiol was achieved on the day of hCG, triggering at 5375 pmol/L, with retrieval of nine oocytes. The patient checked into the emergency room 16 h after the intervention due to severe abdominal pain, paleness, hypotensive with TA 100/60 mm Hg, fainting, tenderness, severe weakness, and dizziness. The ultrasound scan revealed a large amount of fluid surrounding the ovaries and a pocket in the Douglas pouch about 8 cm in diameter. An exploratory laparotomy was conducted 18 h after TVOR and revealed a 2.5 cm laceration on the right ovary with active bleeding and a massive hemoperitoneum of 1600 mL of blood. The right ovary was repaired, and hemostasis was achieved with surgical suturing. The patient received four units of blood, three units of cryoprecipitate, and two units of fresh frozen plasma. The four embryos on day 3 (in cleavage state) were cryopreserved, and 1.5 years later, two embryos were thawed and transferred, resulting in the delivery of twins at 34 weeks’ gestation.

### 3.3. Case Three

A 35-year-old patient was treated in our unit for secondary infertility during tubal obstruction with three previous IVF attempts. She successfully achieved pregnancy and term delivery by caesarean section from the second IVF cycle. Her past medical history included diagnostic laparoscopy for the evaluation of tubal patency, and Hashimoto thyroiditis with hypothyroidism and substitutional therapy in doses of 50 mcg daily, with AMH levels of 3.25 ng/mL. The present IVF cycle was conducted with the GnRh antagonist protocol using 3150 total units of rFSH for COS for 11 days and achieved a peak estradiol level of 5800 pmol/L. She was admitted to the emergency room (ER) 26 h after TVOR intervention with severe diffuse abdominal pain, weakness, dizziness, vomiting, and marked cardiovascular instability with blood pressure values of 90/60 mm and a pulse rate of 100 bpm. An ultrasound scan recorded a large amount of intraperitoneal fluid, particularly around the right ovary, with the pocket in the Douglas pouch at 6–7 cm in diameter. An emergent laparotomy was performed, about 750 mL of blood was drained from the peritoneum, and three or four punctual sites were identified with active bleeding on both ovaries. Hemostasis was achieved with multiple sutures and repairs to both ovaries. The patient received two units of blood. Two cryopreserved embryos on day 3 were not yet thawed and transferred.

### 3.4. Case Four

A 32-year-old patient, gravida 0, para 0, with unexplained infertility, was admitted to our unit after six cycles of IUI for her first IVF attempt. She had no history of any medical conditions or illnesses, and an AMH level of 5.32 ng/mL. Controlled ovarian stimulation employed a GnRh antagonist protocol with 1375 IU of rFSH for 9 days and achieved a peak estradiol level of 9191 pmol/L. Eight hours after TVOR, the patient was admitted to the ER presenting with severe abdominal pain, tenesmus, vomiting, nausea, weakness, shoulder pain, hypotensive with 80/60 mmHg blood pressure values and pulse rate of 95 bpm, with progressive deterioration continuing. The ultrasound scan revealed a large amount of peritoneal fluid–blood, and the dimensions of the right ovary were 55 × 35 mm, with several mixed, predominantly echogenic formations of 22 mm and a 10 cm diameter pocket of fluid in the Douglas pouch. An emergent laparotomy was performed, revealing three or four puncture sites (of punctured follicles) on the right ovary with intermittent active bleeding and about 1000 mL of blood and blood clots. The right ovary was repaired, and hemostasis was achieved with multiple sutures. The patient received four units of blood, one unit of fresh frozen plasma, and two doses of cryoprecipitate in total. The embryo transfer of two cryopreserved and thawed embryos on day 3 was performed 6 months after operation, and a pregnancy was recorded with term delivery of a single baby.

### 3.5. Overall Analysis of Presented Patients

Patients were all in the fourth decade of life and had normal weight (BMI from 18.5 to 24.9 kg/m^2^). None of the patients had any comorbidities, but two had previous surgeries (one had diagnostic laparoscopy and the other had two previous laparotomies, first right salpingectomy due to ectopic pregnancy and right ovary cystectomy and plastic of right fallopian tube). Two had secondary and another primary infertility. Two patients previously also had ART procedures that were uneventful in one case and successful in another case with term delivery (Table 1). 

Controlled ovarian stimulation was performed using GnRh antagonist protocols in three patients while one patient received long GnRh agonists protocol. The triggering was achieved in all cases using hCG or rhCG. Oocyte retrieval was performed in general intravenous anesthesia and using the 17-gauge needle in all cases and negative pressure of 140 mmHg. In average 8.25 ± 2.87 oocytes were retrieved from our patients. No complications and adverse effects of general intravenous anesthesia were recorded.

Symptoms of hemoperitoneum on average occurred at 17.01 ± 7.39 h after TVOR. Only one case became symptomatic (bleeding) in the first 12 h, and a cumulative of three cases (75%) became symptomatic in 24 h after TVOR. All patients were operated as emergency cases through laparotomy. A bleeding ovary was preserved in all patients, and hemostasis was achieved by ovarian suturing. In three cases, right ovary was the source of bleeding, and in one case, involvement of both ovaries was recorded. The operative findings were divided into “diffuse bleeding” and “laceration” or into “bleeding from a puncture site” as a “localized bleeding” (Table 1). 

The average blood volume found intraoperatively was 1137.50 ± 359.11 mL, with hemoglobin levels before surgery at 80.25 ± 4.03 g/dL with hematocrit values of 25.10 ± 2.29% before the surgery. The blood transfusions were performed in all four cases with average number of 6.00 ± 2.94. After uneventful postoperative course, patients were discharged on average on the 6.01 ± 0.81 day. 

Embryo transfers were all delayed, and obtained embryos were cryopreserved. Finally, frozen embryo transfers were performed in three patients of which two achieved pregnancies and gave birth to a healthy child in one case and twins in the second case. The one patient had not thawed her cryopreserved embryos yet. 

### 3.6. Pooled Analysis of All 43 Literature Cases with Ovarian Source Severe Hemoperitoneum

When a thorough literature search was performed, 36 studies presenting patients with severe hemoperitoneum after TVOR were found. No duplication of the studies or use of the same population of patients was found. After screening the titles and abstracts of publications, hemoperitoneum cases due to adjacent vessel lesions (n = 3) were excluded [15,16,17], as well as those from urinary tract lesions (n = 6) [10,31,32,33,34,35]. Out of all full-text articles assessed for eligibility, 26 publications and 7 reports were excluded due to unknown bleeding sources causing hemoperitoneum or unclear presentation of patient data [5,6,26,36,37,38,39]. Two other studies were excluded because they did not report any cases of bleeding or hemoperitoneum as complications after TVOR [4,40]. After a detailed analysis, it was found that only 18 studies reported cases of hemoperitoneum after TVOR caused by ovarian bleeding; therefore, patient data from these studies were included in the pooled analysis [14,18,19,20,21,22,23,24,25,30,41,42,43,44,45,46,47,48]. Finally, the pooled analysis resulted in a total of 43 patients. Patient characteristics, with frequency of investigated parameters, are presented in Table 2 and Table 3. According to the assessed literature, the incidence of severe hemoperitoneum caused mostly by ovarian bleeding ranges from 0.05% to 0.35% [6,26].

Patients had an average age of 31.58 ± 4.93 years and a healthy BMI (average 22 kg/m^2^). Most of the patients were nulliparous and had not undergone previous assisted reproduction procedures (ART). There was a similar number of patients with and without comorbidities, as well as prior surgeries, but significantly more women were not receiving anticoagulant therapy at the time of TVOR. Possible risk factors for ovarian bleeding and hemoperitoneum were reported in just a few studies as a combination of the activated partial thromboplastin time and decreased factor XI and XII concentrations [42], prolongation of the activated partial thromboplastin time [19], intravenous diclofenac treatment before TVOR [41], mild factor VII deficiency and prolonged activated partial thromboplastin time [46], and violent coughing and movement of the body during TVOR [30]. One patient with von Willebrand disease, with a diagnosis established after two episodes of ovarian hemorrhage and peritoneum in two consecutive TVOR interventions, was described after a second bleeding episode [20] (Table 2). 

Prior surgeries in 12 patients included six diagnostic laparoscopies, four cases of ovarian cystectomies (one case after TVOR) and reconstruction of the ovary, and three cases of salpingectomies for different causes. The anticoagulant or anti-aggregated therapy was received by 9 of the 29 patients with reported data, usually due to hematologic diseases and chronic hemodialysis (Table 3).

The long GnRh agonist ovarian stimulation protocol was the most commonly used. Oocyte retrieval was performed under general anesthesia in 50% of the cases using 15–19 gauge needles. On average, 12.51 ± 8.11 oocytes were retrieved, ranging from 0 to 34. Two cases recorded no oocytes retrieved after TVOR [24,46] (Table 2). 

Symptoms of hemoperitoneum occurred 16.63 ± 30.21 h after TVOR. A total of 81.39% (35/43) patients became symptomatic, with signs of bleeding and hemoperitoneum in the first 24 h after TVOR. More than half of the patients (55.81%) became symptomatic in the first 8 h after TVOR, while 10 patients (23.25%) became symptomatic in the first hour post-intervention. The longest period for symptoms of bleeding to develop was 7 days after intervention (Table 3). 

The average interval between TVOR and surgical intervention was 27.19 ± 53.25 h, with intervals ranging from 1 to 264 h (11 days). About 79.05% (34/43) of patients had their surgery less than 24 h after the TVOR. Urgent surgery was performed on 14 patients in the first 8 h after TVOR intervention (32.55%) and on 6 patients (13.95%) 48 h after intervention (Table 3). 

Laparoscopy was the usual surgical approach, and hemostasis was established mostly by electrocoagulation. Table 4 presents data about the type and side of ovarian lesion, surgical intervention, and final hemostatic intervention to manage hemoperitoneum for all 43 patients. The laparoscopic approach was not sufficient for complete hemostasis in four patients, and laparotomy surgery proceeded. In two patients, ovariectomy had to be performed [25,44], and in three cases, wedge or partial ovary resection for hemostasis needed to be implemented [23,30,42]. Hemostasis was achieved in seven cases by combining different methods, such as electrocoagulation and topical agents (adsorbable hemostat) or sutures and adsorbable agents. There was also one case of angiographic uterine artery embolization [46]. 

Two cases of involvement of both ovaries, i.e., bilateral bleeding, were recorded in the study of Battaglia et al. [42], with wedge resection of both ovaries for hemostatic purposes, while one case was noted in our sample that had received simple suturing by laparotomy for hemostasis. The left and right ovaries were presented equally as a source of bleeding in the literature data. The ovary lesions as sources of bleeding were described as “lacerations” in 14 cases, “bleeding from the puncture site” in 15 cases, and “bleeding from the follicle” in 5 cases, meaning “localized” (80.95%), which was more frequent than “diffuse bleeding” or oozing in 9 cases (21.42%) (Table 4).

The time (hours) from TVOR to symptoms and time from TVOR to surgery were significantly longer, with a median of 26 h (range 24–28) and median 41 h (range 30–52) in patients with total ovariectomy, than in patients with ovary-preserving surgery (including partial ovary resection), with median 5.5 h (range 0–76) from TVOR to symptoms and median 11 h (range 1–296) from TVOR to surgery (Table 3).

The average amount of blood drained at surgery from the peritoneal cavity was 1266.91 ± 794.18 mL, ranging from 100 mL with hematoma 10 cm in diameter [47] to 3000 mL as maximum blood volume drained from the abdominal cavity [44]. Average hemoglobin values before surgery were 79.88 ± 17.01 (g/dL) and hematocrit 26.32 ± 6.96%, ranging from 13.50% to 40.00%. There were no postoperative complications in the presented patients. They were discharged on average on postoperative day 5.26 ± 1.92 (Table 3). 

Data about embryo transfer were available for 31 cases (67.44%), including 2 cases with no harvested oocytes. Embryo transfer was delayed in 60% of cases, but in 10 patients, it was performed in the same cycle after surgery, with five pregnancies achieved. Pregnancies were achieved in half of the assessed patients, regardless of whether embryo transfer was fresh or delayed after cryopreservation (Table 2). 

Pooled analysis of incidence regarding hemoperitoneum of ovarian origin after TVOR with surgical treatment for hemostasis showed 0.08% of 28,416 patients [25]. Six studies [14,18,19,21,22,23] evaluated the frequency of hemoperitoneum in their population of patients who underwent IVF procedures, and with the addition of the present study data, this was four cases of 2939 patients (0.13%). Pooled analysis including seven studies showed that the incidence of hemoperitoneum was 0.09% (27/31,355), as presented in Table 5.

## 4. Discussion

Hemoperitoneum after TVOR is a very rare complication, but it has possible serious consequences for infertility patients, their reproductive health, and the outcome of future infertility treatment. In cases of ovarian bleeding causing hemoperitoneum, hemodynamic instability, and hemorrhagic shock, surgical intervention for hemostasis is mandatory, with various interventions to obtain it [18,19,20]. 

We focused on hemoperitoneum as a result of ovarian bleeding with surgery performed for hemostasis, and not from bleeding from other sources, such as injury of blood vessels [15,16,17,39] or from bladder and ureteral injuries [10,31,32,33,34,35]. 

Most of the data regarding the occurrence of hemoperitoneum after TVOR were in the form of case reports and case studies with very heterogeneous data, which makes it difficult to analyze the problem. Only a few studies dealt with the ovary as a source of bleeding after TVOR, while the largest number of studies described the total number of hemoperitoneum instances without specifying the source of bleeding, which is surprising given the seriousness of the complication [5,6,26].

The incidence of hemoperitoneum due to ovarian bleeding in the present pooled analysis was 0.09% of patients, which is similar to the findings of Nouri et al. [25], who reported 0.08%. The incidence of severe hemoperitoneum after TVOR ranges from 0.08% [18] to 0.22% [21]. The large studies evaluated the total incidence of hemoperitoneum, but without a precise source of bleeding identified (presumably a great percentage was of ovarian origin), or the patient’s data not presented separately for each case. Some authors reported that peritoneal bleeding requiring hospitalization was the most common severe complication after TVOR, presenting at 0.23% [6]. Others reported that of the 1,435,108 IVF/ICSI cycles, the incidence of severe hemoperitoneum was 0.08%. This incidence decreased 0.29-fold over a period of nine years, mainly due to the reduction in GnRh agonist protocol use and fresh embryo transfer [26]. Two studies, Ozaltin et al. [40] on 1031 patients and Ludwig et al. [4] on 1166 oocyte retrieval procedures, recorded no single case of bleeding and hemoperitoneum among all complications after TVOR.

No protocols were established for the steps of conducting the procedures for patients presenting with hemoperitoneum caused by ovarian bleeding [25]. Reproductive specialists who perform the TVOR procedure are usually not involved in managing the hemoperitoneum. Urgent care gynecologists, rarely familiar with infertility, make decisions about the type of treatment. The first choice of treatment can be conservative if the patient is hemodynamically stable, with no progressive decline in hemoglobin and hematocrit and no obvious peritoneal volume of fluid (blood) increase. The main goal of conservative observational management is to spare the ovary and avoid ovariectomy [25]. However, the principal question is how long surgery can be delayed without jeopardizing ovary preservation due to underestimation of bleeding severity. Cardiovascular instability with a subsequent drop in hemoglobin and hematocrit values, diffuse abdominal pain, vomiting, nausea, severe weakness, pale skin, hypotension, and tachycardia, regardless of intraperitoneal fluid–blood volume, should be indications for surgical management [20,21,22,23,24,25].

Hematocrit measurement and vital signs are not reliable enough to allow for early bleeding detection, as 100–150 mL blood loss is barely detectable. Abdominal bleeding can be classified by ultrasound, according to the average maximal diameter of the pockets of fluid, as mild (one pocket less than 2 cm maximal diameter), moderate (maximal pocket diameter from 2 to 4 cm), and severe bleeding (maximal pocket diameter more than 5 cm) [49].

The amount of blood found in the peritoneal cavity during surgery is around 100 mL [47] if only the liquid component was evaluated and the amount of blood in a 10 cm diameter periovarian hematoma is not displayed. However, in total, we can presume that the minimum amount is 250 mL [42], and the maximal blood amount 3000 mL [44], with average values of 1266.91 ± 794.17 mL that have been noted in the pooled analysis.

The average blood loss from TVOR intervention for 24 h was 230 mL [27], which is not remarkable and has no clinical significance in an uncomplicated intervention. However, in the case described by Battaglia et al., 250 mL was a sign of hemoperitoneum during surgery when combined with the signs of hemorrhagic shock, which is very close to the definition of normal blood loss during TVOR [42]. This bilateral ovarian involvement case involved a short period between TVOR and surgery (5 h) and required partial resection of both ovaries to achieve adequate hemostasis by laparotomy [42]. 

More than half of the patients became symptomatic in the first 8 h after TVOR (55.81%), while 10 patients (23.25%) became symptomatic in the first post-intervention hour, suggesting serious and profound bleeding. The longest period for developing bleeding symptoms was seven days after TVOR intervention. A total of 18.60% of all patients experienced the first symptoms of bleeding after 24 h; thus, late onset bleeding is not an unusual event. The late onset bleeding can also be attributed to the bleeding originating from ruptured corpora luteal, which are formed in some time after TVOR, and does not mean that all patients are prone to ovariectomy. Presumably, late-onset bleeding is a clinical presentation of ovarian bleeding-caused hemoperitoneum with a large spectrum of events, not necessarily a pathological presentation leading to ovariectomy.

In our data, two patients who underwent ovariectomy had a significantly longer time from TVOR to surgery, 52 and 30 h, than other cases with ovarian-sparing surgery, including three cases with ovarian partial resection patients, who all became symptomatic in the first 12 h [25,44]. Nouri et al. made a similar observation in their pooled analysis of four cases (three cases of ovariectomy and one case of wedge resection) [25]. A too long observational time and postponement of surgery may lead to life-threatening situations and ovariectomy [21,25]. Only one case that ended in ovariectomy became symptomatic after 24 h, or had “late onset” bleeding and experienced an excessively long wait for surgery [21,25]. 

As previously mentioned, “lean women with polycystic ovary syndrome” have been considered a risk factor for ovarian bleeding [21]. In our pooled study, the use of GnRh agonists long protocol reached significance as a predisposing bleeding factor in such patients. The average BMI at pooled analysis was 20.88 ± 2.46, with 87% of women having a BMI of less than 22 kg/m^2^, indicating that they were mostly lean and in a normal weight range. BMI values, number of retrieved oocytes, and TVOR technique did not show enough significance to be regarded as risk factors for ovarian bleeding.

A high number of harvested oocytes, use of the GnRh agonist protocol, lean patients with PCOS, and a high frequency of ovarian hyperstimulation syndrome (OHSS) can predispose patients to ovarian bleeding. Contrary to expectations, swollen hyperstimulated ovaries are fragile, hypervascularized, and prone to bleeding, with easily occurring lacerations. OHSS leads to a hypercoagulant state with higher estrogen levels, which fails to protect patients from ovarian bleeding. Liberty et al. [21] presumed that the leanness of PCOS patients predisposed them to lacerations on fragile, hyperstimulated ovaries, instead of on puncture sites and ovarian lesions, as seen in most patients with hemoperitoneum. Younger age, ovarian stimulation with GnRh agonists, tubal infertility factors, high numbers of retrieved oocytes, and a clinical pregnancy after fresh embryo transfer were associated with an increased risk of hemoperitoneum [26]. A higher number of oocytes retrieved, a longer duration of operative TVOR time, a younger age, and operator experience were significant factors in the occurrence of complications [6].

The GnRh agonist long protocol is often associated with a larger number of harvested cells, higher risk of complications, including OHSS, higher estrogen levels, higher costs for the procedure, long duration, and higher psychological and physical burden for patients [26,50]. Until 2012, this protocol was dominant in most IVF centers, but today, that dominance is held by GnRh antagonist protocols and IVF-friendly protocols with a lower frequency of complications. In our pooled analysis, the GnRh long protocol was the most commonly used protocol for COS (60%) overall, but from 2012 onward, GnRh antagonist protocols took the lead in almost 80% of presented cases [26].

Previous surgery was recorded in 30.2% of the pooled sample of patients and in two of the four patients in our case study. The hypothesis that ovarian scar tissue can disturb ovarian vascularization and the ability to form blood clots for hemostasis did not reach significance for analysis and requires a larger study to be conclusive. Complications in these patients are probably hard to avoid; thus, a senior specialist should perform the intervention.

Abnormalities in the coagulation system were detected in just a few studies (4/43) as a combination of the activated partial thromboplastin time and decreased factor XI and XII concentrations [42], prolongation of the activated partial thromboplastin time [19], intravenous diclofenac treatment before TVOR [41], mild factor VII deficiency, and prolonged activated partial thromboplastin time [46]. Only one case with two consequent episodes of ovarian bleeding and hemoperitoneum after each of the two TVOR attempts was recorded, and after the second incident, a diagnosis of von Willebrand disease was established. [20], suggesting the total effect of coagulation defects on ovarian bleeding was insignificant but revealed the need to perform routine workup with complete blood count and basic coagulation test (prothrombin time and activated partial thromboplastin time tests) routinely before TVOR, which is standard practice in our clinic [51]. Revel et al. [52] stated that 534 coagulation tests were obtained to prevent only one case of bleeding associated with an abnormal result on a coagulation test. When the detection of coagulation abnormalities is positive pre- or post-intervention, consulting a hematologist is necessary, and the transfusion of fresh frozen plasma, concentrated preparations of deficient clotting factor, intravenous globulins, and steroids are recommended [51]. 

Patients receiving anticoagulant therapy before TVOR (18.4%) should be subjected to planned intervention with the collaboration of a hematologist, and the anticoagulant agent must be stopped at least 12–18 h before TVOR and introduced again after the same interval post-intervention. El-Shawarby et al. reported a case with essential thrombocythemia with LMWH application that required salpingo-oophorectomy to achieve hemostasis for ovarian bleeding [44]. Schultz et al. evaluated a case with hemodialysis performed 10 h after the TVOR procedure, along with a systemic anticoagulant, and produced hemoperitoneum 12 h after TVOR, at the time of dialysis [48]. 

Half of the cases in our pooled analysis reported the use of general intravenous anesthesia (50%) and 36.7% intravenous sedation, and 13.3% of TVOR procedures used local anesthesia, with no complications listed apart from one case performed under general intravenous anesthesia with propofol- and pentazocine-induced violent coughing attacks and whole-body movement, producing a laceration of the right ovary [30].

The most frequently used surgical approach was laparoscopy in 60% of all surgeries, and 30% of operations were performed by laparotomy. In four cases, the laparoscopy could not achieve hemostasis; thus, it was converted to a laparotomy. Our clinical protocol, mandatory for cases of hemoperitoneum after TVOR, is to perform laparotomy, and all four of our cases ended with only suturing for hemostasis without any kind of resection of ovarian tissue, even in cases of bilateral ovarian lesions. Laparoscopy is a more demanding operation than laparotomy, and a skillful and trained gynecologic surgeon is required, which is not always possible in an emergency center. Three cases with resection of ovarian tissue were managed with laparotomy [23,42,44], one case of partial ovarian resection was successfully managed with laparoscopy [30], and one case with conversion of laparoscopy to laparotomy ended with unilateral ovariectomy [25]. 

One case in a pooled study group was successfully managed with angiographic uterine artery embolization [46] by an interventional radiologist, which is not available in a gynecological ER, and many do not have equipment and facilities for such interventions in the clinic (tertiary-level university clinic). The authors stated that avoiding the transabdominal route of surgery is favorable for fertility preservation, but disadvantages are more prominent because of the lack of staff and facilities in gynecological clinics and the ER in most tertiary-level clinics.

Intraoperative lesions on the ovary as a source of bleeding (two cases of both ovaries’ involvement) were classified as “localized” (80.95%), in the forms of “laceration” in 14 cases and “bleeding from puncture site” in 15 cases, with “bleeding from the follicle” in 5 cases, and “diffuse bleeding” or “oozing” in 9 cases (21.42%). Similar results were noted by Nouri et al. [25], with 87% localized bleeding and no involvement of both ovaries in their pooled analysis prior to 2014.

Coagulation (mainly laparoscopic) is the most frequently used approach for hemostasis (about 40%), followed by suturing (26%), the sole use of hemostatic agents (5%), and all three methods combined (19%). In 12% of cases, ovarian tissue was removed with partial and wedge ovarian resection, and two cases (12%) involved ovariectomy. Nouri et al. [25] recommended the use of topical hemostatic agents for localized bleeding from the ovary as a supplementary method to electrocoagulation and suturing, which was effective in six cases in their pooled analysis.

Preservation of ovarian reserve is of paramount importance during ovarian surgery, especially in women with infertility. Ovarian suturing is the common therapy for bleeding including those of ovarian origin, but it can result in damage to healthy tissue and an increase in intraovarian pressure in ischemic regions. Bipolar coagulation of the ovarian bleeding site is another effective method of hemostasis but can also potentially harm the surrounding ovarian tissue. Therefore, to avoid damage to healthy ovarian tissue, hemostasis using various topical hemostatic agents has been suggested as a method of choice whenever possible [53,54,55]. Several topical hemostatic agents that exert their effect in a variety of ways are currently available in a range of preparations. The two main categories of topical hemostatic agents are physical agents, which promote hemostasis using a passive substrate, and biologically active agents, which enhance coagulation at the bleeding site. Collagen-based agents in contact with a bleeding surface attract platelets, which adhere to collagen fibrils and degranulate, triggering platelet aggregation and consequently thrombus formation. The mechanism of action of gelatin-based hemostats seems to involve physical surface effects. Oxidized cellulose and oxidized regenerated cellulose act through a number of mechanisms including blood absorption, surface interactions with proteins and platelets, and activation of both the intrinsic and extrinsic coagulation pathways. Fibrin sealants have both hemostatic and adhesive properties while reducing the formation of adhesions, which can even enhance wound healing. Relatively recent agents are those based on polysaccharides, albumin, glutaraldehyde, and inorganic agents. Studies have shown that using hemostatic sealants has a positive effect on the ovarian reserve compared to the use of bipolar coagulation and ovarian suturing, as these patients had better levels of anti-Mullerian hormone and antral follicular count postoperatively [53,54,55]. 

Embryo transfer in a fresh cycle immediately after surgery was performed in 33.3% of cases (10 of 31 patients with available data about embryo transfer and 29 patients with possible embryo transfer). The embryos were cryopreserved in 60% of cases and thawed and transferred a few months later. All cases of fresh embryo transfer after surgery in the same cycle were reported until 2011, and despite everything, had quite a good pregnancy rate of 50% [23]. In our sample, there were no cases of fresh embryo transfer. The pregnancy rate of frozen embryo transfer (FET) was also 50%. The overall rate of pregnancy in the pooled analysis, regardless of whether embryo transfer was fresh or frozen, was 50%; thus, the reproductive potential after surgery appeared to be preserved.

After the year 2011, the doctrine of embryo transfers gradually changed, and “freeze all” came into practice without compromising success rates. Kuroda et al. [26] proposed that the embryos of patients with intraperitoneal hemorrhage should be frozen. There is no justification from a clinical point of view regarding the success of frozen embryo cycles, and no financial reasons to conduct the transfer in a fresh cycle immediately after operation.

This serious complication of TVOR, hemoperitoneum originating from ovarian bleeding, is potentially life threatening and, fortunately, remains very rare. There are about 39 cases published, mainly in the form of case reports and case series. It is possible that the total number of cases of this pathology is underestimated. We found only four cases in a 12-year period with complete documentation, in which TVOR was conducted in our IVF center and patients came into the ER in our clinic. There is a potential selection bias in this case because general incidence is not covered, as patients with mild symptoms of hemoperitoneum and observation for a few hours without admission to hospital were not included. In addition, patients who went to other gynecology emergency facilities for treatment complications were not reported. The data should be collected from the whole state, as this would result in more accurate numbers of this pathology. The most serious consequence revealed in this investigation is that hemostatic treatment ended in ovariectomy. This is disastrous for women seeking infertility treatment and indicates failure for the reproductive doctor. To date, only two cases of ovariectomy have been recorded, which is insufficient to draw conclusions. We encourage other authors to publish their case reports and case series to obtain a sufficient number for statistical analysis and risk factor identification. Often, reproductive specialists are not interested in reporting such complications of the IVF process. Cases with adverse outcomes are more likely to be published than conservatively managed cases of mild hemoperitoneum; thus, these two cases of ovariectomy may be inflated compared to the real and overlooked total numbers of complications. 

## 5. Conclusions

The severe complications of transvaginal oocyte retrieval, such as ovarian hemorrhage and hemoperitoneum, that necessitate surgery, are generally rare, with a frequency of 0.09% from pooled analysis of 31,355 procedures of TVOR. Nevertheless, they are still one of the gravest TVOR complications that should always be kept in mind. 

Conservative minimally invasive management should be attempted if the patient is cardio-vascularly stabile. In case of hematoperitoneum, the type of surgery and intervention leading to hemostasis should be personalized to each patient and based on surgeon’s experience to avoid adverse outcome as ovariectomy. 

Embryos collected from these cycles should be cryopreserved and used later for frozen embryo transfer.

Further publications on larger samples of this rare event are needed in order to identify the real incidence of this iatrogenic pathology and to define the possible protocols of its treatment. Patients should be given the correct information about complications of the oocyte retrieval procedures, their incidence and management, and possible consequences.

## Figures and Tables

**Table 1:** Investigated parameters of patients from our Clinic.

Parameters	Patient 1	Patient 2	Patient 3	Patient 4
Patients age	38	34	35	32
Body Mass Index (kg/m^2^)	24.1	19.72	22	20.1
Previous parity	0	0	1	0
Previous assisted reproduction	yes 1×	no	yes 3×	no
Previous surgery	yes	no	yes	no
Comorbidities	no	no	no	no
Anticoagulation therapy	no	no	no	no
Stimulation protocol	GnRh agonists	GnRh antag	GnRh antag	GnRh antag
Retrieved oocytes number	6	9	6	12
Oocyte retrieval needle (G-gauge)	17	17	17	17
Anesthesia for oocyte retrieval	General iv	General iv	General iv	General iv
Time to symptoms (h)	18	16	26	8
Time to surgery (h)	20	18	29	9
Surgical approach	laparotomy	laparotomy	laparotomy	laparotomy
Hemostasis	suturing	suturing	suturing	suturing
Blood volume in abdomen (mL)	1200	1600	750	1000
Hemoglobin before surgery (g/dL)	82	78	85	76
Hematocrit (%) before surgery	27	24	27	22.4
Transfusions	6	9	2	7
Hospitalization days	6	7	6	5
Embryo transfer	postponed	postponed	postponed	postponed
Frozen embryo transfer	yes	yes	no	yes
Achieved embryo number	4	5	2	8
Pregnancy achieved	no	live birth	no	live birth

Legend: Antag-antagonists; GnRh-Gonadotropine Releasing hormone; iv-intravenous; mL-millimeters; h-hours; kg-kilograms, m^2^-square meters.

**Table 2:** Descriptive data of pooled sample (patients from our Clinic and previous studies).

Parameters	Minimum	Maximum	Mean	Standard Deviation	*p*
Patients age	20.00	41.00	31.58	4.93	0.001
Body Mass Index (kg/m^2^)	17.80	27.60	20.88	2.46	0.001
Retrieved oocytes number	0.00	34.00	12.51	8.11	0.001
Achieved embryo number	0.00	18.00	5.15	4.84	0.001
Oocyte retrieval needle gauge	15.00	19.00	17.12	0.74	0.001
Time to symptoms (h)	0.00	168.00	16.63	30.21	0.001
Time to surgery (h)	0.00	264.00	27.19	53.25	0.002
Blood volume in abdomen (mL)	100.00	3000.00	1266.91	794.17	0.001
Hemoglobin before surgery (g/dL)	47.00	142.00	79.88	17.01	0.001
Hematocrit (%) before surgery	13.50	40.00	26.32	6.96	0.001
Transfusions	0.00	12.00	3.82	3.24	0.001
Hospitalization days	2.00	10.00	5.26	1.92	0.001

Legend: Kg-kilograms; m^2^-square meters; h-hours; mL-milliliters; g-grams, Dl-deciliters.

**Table 3:** Frequency of investigated parameters of pooled sample (patients from our Clinic and previous studies).

Parameters		Frequency	Percent	*p*
Previous parity	0	28	87.5	0.001
	1	3	9.4	
	3	1	3.1	
Previous assisted reproduction	0	12	50.0	0.001
	1	9	37.5	
	more	3	12.6	
Previous surgery	no	15	55.6	0.564
	yes	12	44.4	
Comorbidities	no	12	35.3	0.142
	hematologic	8	23.5	
	pelvic inflammation	3	8.8	
	PCO Syndrome	5	14.7	
	other	6	17.6	
Anticoagulation therapy	no	20	69.0	0.041
	yes	9	31.0	
Stimulation protocol	natural	1	3.3	0.001
	long GnRh agonists	18	60.0	
	GnRh antagonists	11	36.7	
Anesthesia	local	4	13.3	0.045
	IV sedation	11	36.7	
	general	15	50.0	
Time to symptoms	≤8 h	25	58.1	0.002
	8 to 24 h	10	23.3	
	≥24 h	8	18.6	
Surgical approach	laparoscopy	25	58.1	0.001
	laparotomy	13	30.2	
	both	4	9.3	
	other	1	2.3	
Hemostasis	coagulation	16	37.2	0.001
	sutures	11	25.6	
	agents	2	4.7	
	all or other	9	20.9	
	ovary resection	3	7.0	
	ovariectomy	2	4.7	
Embryo transfer	performed	10	33.3	0.002
	postponed	18	60.0	
	no	2	6.7	
Frozen embryo transfer	no	11	42.3	0.433
	yes	15	57.7	
Pregnancy achieved	no	12	50.0	1.000
	yes	12	50.0	

Legend: Gnrh-gonadotropine releasing hormone; PCO-polycystic ovary.

**Table 4:** Type of ovarian lesion, surgical management and hemostatic intervention after TVOR: a pooled analysis.

Studies	Case No	Left/Right Ovary	Intervention	Ovarian Lesion-Type	Surgical Intervention-Hemostasis
Bennett et al. (1993) [14]	1	n/a	laparotomy	multiple points in capsule	suturing
Dicker et al. (1993) [18]	1	n/a	laparotomy	laceration	electrocoagulation, suturing
	2	n/a	laparoscopy	puncture site	electrocoagulation
	3	n/a	laparoscopy	puncture site	electrocoagulation
Govaerts et al.(1998) [19]	1	n/a	laparoscopy	puncture site	electrocoagulation
	2	n/a	laparoscopy	puncture site	electrocoagulation
	3	n/a	laparoscopy	puncture site	electrocoagulation
Battaglia et al. (2001) [42]	1	both	laparotomy	diffuse leakage both	partial resection both
El-Shawarby et al. (2004) [44]	1	right	laparotomy	diffuse, general oozing	right salpingo-oophorectomy
Moayeri et al. (2007) [20]	1	left	laparoscopy	laceration	electrocoagulation
	2	left	laparoscopy	oozing	electrocoagulation
Bandyopadhyay and Kay. (2010) [41]	1	left	laparoscopy/laparotomy	profuse, rupture ovary	suturing
Liberty et al. (2010) [21]	1	n/a	laparoscopy/laparotomy	tear, active bleeding	suturing, adsorable hemostat
	2	n/a	laparoscopy	tear, active bleeding	electrocoagulation, topical hemostat
	3	n/a	laparoscopy	puncture site	electrocoagulation
	4	n/a	laparoscopy	tear, active bleeding	electrocoagulation, topical hemostatic
	5	n/a	laparoscopy	tear, active bleeding	electrocoagulation
	6	n/a	laparoscopy	tear, active bleeding	topical hemostatic agents
	7	n/a	laparoscopy	oozing puncture site	electrocoagulation, adsorable hemostst
Zhen et al. (2010) [22]	1	left	laparotomy	puncture site	suturing
	2	right	laparotomy	two puncture sites	suturing
	3	right	laparotomy	laceration	suturing
	4	right	laparotomy	puncture sites	suturing
	5	left	laparoscopy	two puncture sites	electrocoagulation
Aragona et al. (2011) [23]	1	left	laparoscopy	puncture site	electrocoagulation
	2	right	laparoscopy	puncture site	electrocoagulation
	3	left	laparotomy	diffuse	wedge resection
	4	left	laparoscopy	laceration	electrocoagulation
Kart et al. (2011) [46]	1		angiographic uterine artery embolization	extravasation ovary	angiographic uterine artery embolization
Chatrian et al. (2012) [43]	1	left	laparoscopy	puncture site	electrocoagulation, topic hemostatic agent
Mashiach et al. (2013) [24]	1	left	laparoscopy	infundibulopelvic ligament and ovary laceration	electrocoagulation
	2	right	laparoscopy	tear of capsule	electrocoagulation
Nouri et al. (2014) [25]	1	left	laparoscopy/laparotomy	diffuse	unilateral ovariectomy
	2	right	laparoscopy/laparotomy	diffuse	electrocoagulation, topical hemostat
	3	right	laparoscopy	puncture site	topical hemostat agents
Huang et al. (2021) [45]	1	left	laparoscopy	puncture site	electrocoagulation
Okoschi et al. (2021) [30]	1	right	laparoscopy	laceration	partial ovary resection
Sachdeva et al. (2022) [47]	1	left	laparoscopy	puncture site	suturing
Schultz et al. (2022) [48]	1	n/a	laparoscopy	diffuse, millimeter sites	electrocoagulation
Stojnic et al. present	1	right	laparotomy	laceration	suturing
	2	right	laparotomy	laceration	suturing
	3	both	laparotomy	diffuse, 4 sites each	suturing
	4	right	laparotomy	diffuse, 5 sites	suturing

Legend: n/a-not available.

**Table 5:** Pooled analysis of eight studies that investigated the frequency of ovarian bleeding caused hemoperitoneum after transvaginal oocyte retrieval.

Studies	Hemoperitonuem from Ovarian Bleeding (n)	Number Total TVOR (n)	Frequency of Hemoperitoneum (%)
Bennett et al. (1993) [14]	1	2670	0.04
Dicker et al. (1993) [18]	3	3656	0.08
Govaerts et l.(1998) [19]	3	1500	0.20
Liberty et al. (2010) [21]	7	3241	0.22
Zhen et al. (2010) [22]	5	10,251	0.05
Aragona et al. 2011) [23]	4	7098	0.06
Stojnic et al., present	4	2939	0.14
Pooled analysis	27	31,355	0.09

Legend: TVOR-transvaginal oocyte retrieval.

## Data Availability

The data presented in the study are available upon request from the corresponding author, but are not publicity available as there are personal patients findings.
